# Cellular and humoral immune responses associated with protection in sheep vaccinated against *Teladorsagia circumcincta*

**DOI:** 10.1186/s13567-021-00960-8

**Published:** 2021-06-16

**Authors:** Cynthia Machín, Yolanda Corripio-Miyar, Julia N. Hernández, Tara Pérez-Hernández, Adam D. Hayward, Harry W. Wright, Daniel R. G. Price, Jacqueline B. Matthews, Tom N. McNeilly, Alasdair J. Nisbet, Jorge F. González

**Affiliations:** 1 Facultad de Veterinaria, Instituto Universitario Sanidad Animal y Seguridad Alimentaria, Universidad de Las Palmas de Gran Canaria, Arucas, Spain; 2 Moredun Research Institute, Edinburgh, UK; 3 Roslin Technologies, Edinburgh, UK

**Keywords:** Nematode vaccine, Genetic resistance, Immune response, Immunoglobulins, Globule leukocytes, *Teladorsagia circumcincta*, Sheep, Cellular response

## Abstract

Due to increased anthelmintic resistance, complementary methods to drugs are necessary to control gastrointestinal nematodes (GIN). Vaccines are an environmentally-friendly and promising option. In a previous study, a *Teladorsagia circumcincta* recombinant sub-unit vaccine was administered to two sheep breeds with different levels of resistance against GIN. In the susceptible Canaria Sheep (CS) breed, vaccinates harboured smaller worms with fewer eggs in utero than the control group. Here, we extend this work, by investigating the cellular and humoral immune responses of these two sheep breeds following vaccination and experimental infection with *T. circumcincta*. In the vaccinated CS group, negative associations between antigen-specific IgA, IgG_2_ and Globule Leukocytes (GLs) with several parasitological parameters were established as well as a higher CD4^+^/CD8^+^ ratio than in control CS animals, suggesting a key role in the protection induced by the vaccine. In the more resistant Canaria Hair Breed (CHB) sheep the vaccine did not significantly impact on the parasitological parameters studied and none of these humoral associations were observed in vaccinated CHB lambs, although CHB had higher proportions of CD4^+^ and CD8^+^ T cells within the abomasal lymph nodes, suggesting higher mucosal T cell activation. Each of the component proteins in the vaccine induced an increase in immunoglobulin levels in vaccinated groups of each breed. However, levels of immunoglobulins to only three of the antigens (Tci-MEP-1, Tci-SAA-1, Tci-ASP-1) were negatively correlated with parasitological parameters in the CS breed and they may be, at least partially, responsible for the protective effect of the vaccine in this breed. These data could be useful for improving the current vaccine prototype.

**Supplementary Information:**

The online version contains supplementary material available at 10.1186/s13567-021-00960-8.

## Introduction

The protective immune response in sheep against *Teladorsagia circumcincta* infection has been studied extensively and is associated with lower worm egg production and parasite burden, as well as shorter and less fecund female adult nematodes [1, 2]. A predominantly Type-2 response has been implicated in this immune response, recruiting eosinophils, mast cells, globule leukocytes (GL), with concomitant production of parasite-specific IgA, IgG and IgE as well as some cytokines such as IL-4 and IL-5 [3]. Associations between the nematode specific IgA levels and reduced worm length and worm eggs in utero (EIU) in *T. circumcincta* infections have been reported [1, 2, 4, 5].

Until recently, control of *T. circumcincta* has been focused on the use of chemotherapeutics, but nematode resistance against anthelmintics has made this approach unsustainable [6]. Due to this issue, it is important to find alternative control measures, such as vaccination; understanding the mechanisms underlying the protective immune response is essential for the development of a successful vaccine [7]. Although finding a successful prototype recombinant vaccine against gastrointestinal nematodes (GINs) has been a difficult task, a recombinant sub-unit vaccine against *T. circumcincta* was recently developed and has shown repeated effectiveness [7–10]. Previous successful prototype vaccines against GIN were only effective in their native forms, preventing global distribution and commercialisation. This recombinant prototype has some features which could overcome this hurdle but, as with other prototype recombinant vaccines against GINs, repeated trials have shown variability in protective response [9]. In addition, the combination of 8 antigens in a single vaccine makes its production prohibitively expensive and simplifying the vaccine would be very desirable.

In a recent study [11], parasitological data was collected for evaluating the effects of this vaccine in two sheep breeds, the Canaria Sheep (CS) and the Canaria Hair Breed (CHB), which have different susceptibilities to *T. circumcincta*. Whereas in vaccinated CS animals there were significant reductions in worm length and numbers of worm intrauterine eggs, vaccination in CHB also induced reductions in parasitological variables, but these were not statistically significant. Here, as an extension of this study, we report the main immune responses that were found in vaccinated sheep. Data obtained would help increase the efficacy of the prototype vaccine and bring it nearer to market.

## Materials and methods

### Animals and experimental design

The experimental design was previously described in details in [11]. Briefly, 24 male lambs of CHB and another 24 male lambs of CS, all of them weaned, were dewormed and kept worm-free until they were six months-old. These animals were randomly selected, within each breed, to establish 4 groups (CHB-vaccine, CHB-control, CS-vaccine and CS-control) of 12 lambs in separated pens. One animal from the vaccinated group of CHB died for non-related causes before the experimental procedures began.

Vaccinated groups (CHB-vaccine and CS-vaccine) were injected subcutaneously (3 doses, on days 0, 21 and 42) with 400 µg of antigens [given as 2 injections: one with cathepsin F-1 (Tci-CF-1), a 20 kDa protein of unknown function (Tci-ES20), activation-associated secretory protein-1 (Tci-ASP-1), a homologue of a protective antigen from *Ancylostoma caninum* (Tci-SAA-1), macrophage migration inhibitory factor-1 (Tci-MIF-1), calcium-dependent apyrase-1 (Tci-APY-1) and a TGFβ homologue (Tci-TGH-2) in PBS; and the other with astacin-like metalloproteinase-1 (Tci-MEP-1) in PBS/Urea] plus 10 mg Quil A (*Vax Saponin, Guinness Chemical Products Ltd*) [8]. Control groups received the same volume of urea/PBS/10 mg Quil A each time. At the same time as the final immunisation (day 42), a “trickle infection” protocol was carried out where all animals were inoculated with 2000 third stage larvae (L3) of *T. circumcincta* three times per week for 4 weeks until day 68.

Faecal egg counts (FEC) were performed using modified McMaster technique [12] three times per week from day 56 after the start of immunisations, until the end of the experiment. At the end of the trial (days 82–85), lambs were euthanised, and adult and immature worms from aliquots of the abomasal content were obtained, counted and measured following standard techniques [11].

### Enzyme-linked immunosorbent assay

Animals were bled from the jugular vein on day 77 following the first immunisation, four days prior to post-mortem. These samples were collected in silicone-coated tubes (Gel + Clot Act. VenoSafe™, TERUMO) for blood serum and were refrigerated at 4 °C for at least 30 min. Then, they were centrifugated at 1164 × *g* (Mixtasel, Selecta) and the serum obtained kept at -20 °C until use. ELISA was performed to determine levels of IgA, IgG_1_ and IgG_2_ in sera from individual sheep following a previously published protocol [13] with minor modifications. Microplates (*Corning*®) were coated overnight at 4 °C with 5 μg/mL of antigen (L4 and adult stage of *T. circumcincta*; or individual recombinant antigens from the vaccine) in carbonate buffer (50 µL per well). All incubations were done at 37 °C. After three washes with Phosphate Buffered Saline (PBS) + 0.05% Tween 20 and blocking for 1 h with 3% bovine serum albumin in PBS, samples diluted at 1:200 in Tris Buffered Saline containing 0.05% Tween®20 (TBST) were incubated for 1 h (each sample was performed in duplicate). Following a further wash, mouse anti-sheep IgA (1:8000 in TBST for parasite antigens and 1:4000 for vaccine antigens), IgG_1_ or IgG_2_ (clones McM1 and McM3 respectively, at 1:1000 in TBST) were added and incubated for 1 h. Plates were then washed and polyclonal rabbit anti-mouse immunoglobulins conjugated to HRP (*Dako*) added at 1:1000 and incubated for 1 h. After a final wash step, 100 µL of O-phenylene-diamine dihydrochloride substrate (*Sigma Fast OPD Tablets*) was added to each well (final concentrations of 0.4 mg/ml OPD, 0.4 mg/ml urea hydrogen peroxide, and 0.05 M phosphate-citrate) and the reaction stopped by adding 25 µL of 2 M sulphuric acid. Finally, Optical Density (OD) values were read at 490 nm (Multiskan Ascent). A serum sample from a non-infected animal was included as a negative control, while the positive control sera was obtained from trickle infected vaccinated animals from this trial. The optical densities were transformed into an optical density index (ODI) for each animal using the formula: ODI = (mean OD—mean negative OD)/(mean positive OD—mean negative OD) [14], adding 1.0 to all values to avoid negative data for statistical analysis.

### Lymphocyte stimulation assays

Abomasal lymph node (ALN) cells were collected aseptically at post-mortem (days 82–85) in cold transport wash media [TWM (HBSS w/o Ca^2+^ or Mg^2+^ supplemented with 2% heat inactivated foetal calf serum (FCS), 100U/mL penicillin, 100 μg/mL streptomycin and 2% gentamicin, all from Sigma)] and kept on ice until processing. In order to obtain single cell suspensions from the ALN, lymph nodes were rinsed with fresh cold TWM, placed in a petri dish and cleaned from excess adipose tissue. Around 3 mL of fresh TWM was added to the petri dish, and tissue was then dissociated using a sterile syringe plunger. Cell suspension was then filtered through 70 μm cell strainers to remove large clumps and tissue fragments. Samples were allowed to settle for 10 min and any debris/excess cells discarded. Following two washes with TWM, cell suspensions were frozen in freezing media (FBS + 10% cell culture grade DMSO (Sigma)) and stored in liquid nitrogen until processed. Prior to stimulations, frozen cells were gently defrosted in a water bath and warm complete media (RPMI-1640 medium supplemented with 10% FCS, 2 mM L-glutamine, 100 U/mL Penicillin, 100 μg/mL Streptomycin, 0.5% of gentamicin and 50 μM 2-mercaptoethanol (all from Sigma)) gently added to the vials. Cells were then washed twice with fresh media and re-suspended at 2 × 10^6^ cells/mL. Lymphocyte stimulation assays (LSA) were carried out by incubating 2 × 10^5^ ALN cells in triplicate with equal volumes of PBS (negative control), ConA (positive control), soluble L4 *T. circumcincta* or Adult *T. circumcincta* antigen (all stimulants at 5 μg/mL final concentration), in a total volume of 200 μL, at 37 °C with 5% CO_2_ in air for 5 days. After 4 days, 50 µL of media from each replicate was collected and stored at −20 °C for cytokine measurements and replenished with fresh complete media containing methyl-^3^H thymidine (0.5 μCi per well). Proliferation was measured by the incorporation of methyl-^3^H thymidine during the final 18 h of culture and expressed as Stimulation Index (SI) by dividing the proliferation of samples incubated with *T. circumcinta* antigen by that from PBS controls.

### Cytokine ELISA

Capture ELISAs were performed to examine the antigen specific secretion of interferon (IFN)-γ Interleukin (IL)-4 and IL-17A by ALN. All incubations were carried out at room temperature unless stated otherwise. Interleukin-4 and IFN-γ were quantified using commercial ELISA kits according to the manufacturer’s instructions (MABTECH AB, Augustendalsvägen, SE, Sweden). For the quantification of IL-17A, polyclonal rabbit anti-bovine IL-17A antibodies were used alongside bovine recombinant protein (all from Kingfisher Biotech, Inc., St. Paul, MN). Washing steps for all ELISAs were performed 6 times with 350 μL washing buffer (Phosphate Buffered Saline (PBS) + 0.05% Tween®20) using a Thermo Scientific Wellwash™ Versa (ThermoFisher). Briefly, high-binding capacity ELISA plates (Immunolon™ 2HB 96-well microtiter plates, ThermoFisher) were incubated with coating antibodies overnight at 4 °C. Plates were then washed and blocked for 1 h with PBS containing 0.05% Tween®20 (Sigma, UK) and 0.1% BSA Bovine Serum Albumin (BSA, Sigma, UK). Following a further washing step, 50 μL of supernatants or standards were added in duplicate for 1 h. Subsequently, plates were washed, and detection antibodies added for 1 h. This was followed by washing and addition of Streptavidin-HRP (Dako, Agilent, Santa Clara, USA) for 45 min. After the final washing step, 50 μL of SureBlue TMB substrate (Insight Biotechnology, London, UK) was added and the reaction was stopped by the addition of equal volume of TMB stop solution (Insight Biotechnology, London, UK). Absorbance values were read at OD 450 nm using a Sunrise™ microplate reader (Tecan, Männedorf, CH, Switzerland). In order to quantify the cytokines of interest, samples were analysed 1:2 for IFN-γ or neat for IL-4 and IL-17A. All values were blanked corrected and concentrations determined from the standard curves included in all plates which were constructed using 7 serial dilutions of recombinant cytokines ranging from 400 to 6.25 pg/mL for IFN-γ (MABTECH AB); 2000 to 62.5 pg/mL for IL-4 (MABTECH AB) and 1500 to 23.43 pg/mL for IL-17A (Kingfisher).

### Phenotyping of ALN cells by flow cytometry

Single colour flow cytometry was carried out in resuscitated ALN cells using the monoclonal antibodies detailed in Additional file 1 at pre-optimised concentrations. All incubations were carried out at room temperature and protected from light. Briefly, 5 × 10^5^ ALN cells per antigen/no antibody control/secondary only control were blocked with 200 μL of 20% heat inactivated normal goat serum (BioRad) for 15 min. Following centrifugation, supernatants were discarded and cells incubated with their corresponding antibody or FACS buffer (PBS + 5% heat inactivated FCS plus 0.02% sodium azide) for controls for 20 min. Cells were then washed twice with FACS buffer and incubated with anti-mouse IgG (H + L) conjugated to Alexa Fluor® 647 (Invitrogen, Life Technologies, USA) for 20 min. After two washes, cells were resuspended in dead cell stain Sytox Blue (Invitrogen, Life Technologies, USA) prior to acquisition. A minimum of 25 000 events were acquired using a MACSQuant® Analyzer 10 (Miltenyi Biotech, Germany) and analysed using FlowJo vX for Windows 7. Dead cell and doublet cell discrimination was performed during all analysis.

### Histochemistry and morphometric analysis

Abomasal tissue samples (approximately 2 cm × 2 cm) were collected at post-mortem from the antropyloric region for histological and immunohistochemistry studies, performed as described previously [15]. Eosinophil and globule leukocyte (GL) counts were performed in abomasal tissue stained with haematoxylin and eosin (Additional file 2), while toluidine blue staining was used to determine mast cell numbers. For each animal sample, cells were counted in at least 20 randomly selected fields of 0.06 mm^2^ (0.245 mm × 0.245 mm) at 400 × magnification with an optical microscope (Olympus CX31) and expressed as cell/mm^2^. Eosinophils and mast cells were counted in selected fields in the lamina propria corresponding to the lower third of the abomasal mucosa. Globule leukocytes were counted in the upper third of the mucosa.

The tissue samples taken for immunohistochemistry were used to identify and count the following cell populations: CD4^+^, CD8^+^, γδTCR^+^, MHCII^+^, CD45RA^+^ and Galectin-14^+^ (Additional file 1) [15]. Cells were counted in 20 fields located in upper and lower third of the mucosa and were expressed in cells/mm^2^, as described above (Additional file 2).

### Statistical analysis

*IBM SPSS Statistics version 24.0* software was used for statistical analysis of parasitological, immunohistochemical and serum antibody data. Differences in FEC, cumulative FEC, worm counts, worm length and EIU were estimated as described in [11]. The data relative to specific immunoglobulin levels in serum were analysed using generalised linear models (GENLIN), using the estimation method Newton–Raphson and Pearson Chi-square scale. The general linear model (GLM) univariate was used for cellular counts, taking LSD (Least Significance Difference) test as reference.

Analyses for immune cell phenotypes were performed using RStudio package version 1.1.456 (R Core Team 2019) as follows: flow cytometry and proliferation data was analysed using two-way ANOVA to test for differences between breeds, vaccination groups (control, vaccinated), and their interaction. Cytokine data was analysed using a GLM as follows: IFN-γ and IL-4 data was log-transformed prior to analysis to satisfy normality of residuals and homoscedasticity. Both cytokines were fitted with effects of vaccine, breed and antigen-stimulation (treatment), as well as two-way interactions and the three-way interaction. Models were simplified by stepwise deletion based on Wald F-tests.

Spearman’s rank correlation coefficient was used for studying correlations between immunological, cellular and parasitological variables.

For all analyses, probabilities of *p* < 0.05 were considered statistically significant. One animal from CS-control group was excluded from all statistical analyses because the abomasal wash sample was lost, meaning no worm burden data was available. Figures were produced with RStudio package version 1.1.456.

## Results

### Parasitological data

Parasitological data from this trial was previously presented in [11]. Briefly, vaccination induced a statistically significant reduction in worm length and EIU in the vaccinated CS group when compared to the control (adjuvant-only) CS group (Table 1).

**Table 1:** **Parasitological data from Canaria Sheep (CS) and Canaria Hair Breed (CHB) sheep vaccinated with a prototype recombinant sub-unit** ***Teladorsagia circumcincta* vaccine and subsequently challenged with** ***T. circumcincta***

Group	Cumulative FEC	Worm burden	Worm length (mm)	EIU
CS Vac	2861 ± 737	3606 ± 803	9.10 ± 0.10**	16.39 ± 0.77**
CS Con	4372 ± 1021	4148 ± 761	9.87 ± 0.06**	23.67 ± 0.87**
CHB Vac	720 ± 205	1171 ± 459	8.82 ± 0.12	14.65 ± 0.73
CHB Con	1157 ± 503	2052 ± 548	9.07 ± 0.09	16.03 ± 0.67

Values shown are Means (± SEM) and values with statistically significant differences within breeds are represented with “**” at *p* < 0.01. Abbreviations: Vac:  Vaccinated, Con:  Control (adjuvant-only), FEC:  Faecal egg count; EIU: Eggs in utero. Data obtained from González et al. [11].

### Measurement of antibody responses to parasite and vaccine antigens in serum

In order to evaluate if the vaccine has induced some variations in the humoral immune response against this worm, the response to the L4 stage -target of the protective immunity in sheep- was firstly analysed in vaccinated and non-vaccinated infected groups of both breeds. Immunoglobulin A was the only isotype for which a statistically significant increase, associated with vaccination, against L4 antigen was observed, in the vaccinated CHB group (*p* < 0.01) compared to the CHB control group (Table 2). Negative correlations between levels of L4 extract-specific serum IgA and IgG_2_ and worm length or EIU were observed in vaccinated groups, being only statistically significant in the CS vaccine recipients. Levels of L4 extract specific IgG_2_ were also negatively associated with worm burden and cumulative FEC in CS vaccine recipients (Table 2).

**Table 2:** **Levels of serum immunoglobulins against extracts of the fourth larval stage (L4) of *Teladorsagia circumcincta* in Canaria Sheep (CS) and Canaria Hair Breed (CHB) sheep vaccinated with a prototype recombinant sub-unit** ***T. circumcincta* vaccine and subsequently challenged with *T. circumcincta***

Isotype	Group	Mean ± SEM	Cumulative FEC	Worm burden	Worm length	EIU
Correlation						
IgA	CS	1.189 ± 0.054	−0.223	0.016	−0.333	−0.267
	CS Vac	1.160 ± 0.066	−0.371	−0.545	−0.776**	−0.615*
	CS Con	1.220 ± 0.089	0.027	0.542	−0.023	0.255
	CHB	1.335 ± 0.073	−0.077	−0.335	0.065	−0.007
	CHB Vac	1.509 ± 0.124**	− 0.545	−0.600	0.042	−0.212
	CHB Con	1.175 ± 0.055**	0.263	0.259	0.203	0.224
IgG1	CS	1.547 ± 0.057	−0.033	0.191	0.050	0.120
	CS Vac	1.508 ± 0.151	0.224	0.084	−0.238	−0.182
	CS Con	1.590 ± 0.145	−0.164	0.436	0.164	0.564
	CHB	1.305 ± 0.103	0.107	−0.179	0.259	0.234
	CHB Vac	1.312 ± 0.063	−0.227	−0.191	0.261	0.261
	CHB Con	1.300 ± 0.095	0.305	−0.042	0.196	0.245
IgG2	CS	1.241 ± 0.047	−0.158	0.037	−0.190	−0.239
	CS Vac	1.234 ± 0.070	−0.594*	−0.594*	−0.867**	−0.825**
	CS Con	1.249 ± 0.064	0.227	0.782**	0.291	0.473
	CHB	1.205 ± 0.050	0.345	0.012	0.448*	0.655**
	CHB Vac	1.255 ± 0.092	0.327	0.073	0.406	0.733*
	CHB Con	1.160 ± 0.046	0.261	0.042	0.483	0.578*

Antibody levels are expressed as optical density index (ODI) value + 1 (ODI + 1). Associations are expressed as Spearman’s correlation coefficient. Statistically-significant differences within breeds for a specific isotype and antigen and significant correlations are represented with “*” at *p* < 0.05 and “**” at *p* < 0.01.

Specific IgA, IgG_1_ and IgG_2_ levels in serum against all recombinant antigens were significantly higher in the vaccinated sheep than in control sheep (Table 3, Additional files 3, 4, 5, 6, 7 and 8). Specific serum IgA levels against all studied recombinant antigens were very similar within breeds, though levels of IgA specific for Tci-CF-1 were significantly lower than those against Tci-APY-1 in vaccinated CS animals (Additional files 3 and 4). Greater variability in vaccinated groups was observed in the levels of specific IgG_1_ and IgG_2_ against these recombinant proteins within breeds (Table 3, Additional files 3, 4, 5, 6, 7 and 8). In vaccinated CS, IgG_1_ levels specific for Tci-CF-1 and Tci-SAA-1 were similar and were significantly higher than IgG_1_ levels against the other recombinant proteins, although IgG_1_ against Tci-MEP-1 was not significantly lower than Tci-SAA-1-specific IgG_1_. IgG_1_ levels against Tci-MEP-1 were significantly higher than IgG_1_ levels specific for Tci-ES20 and Tci-APY-1. On the contrary, IgG_1_ specific for Tci-MIF-1 was significantly lower than IgG_1_ specific for Tci-ASP-1, Tci-ES20, Tci-MEP-1 and Tci-TGH-2 (Additional file 5). In vaccinated CHB animals, levels of IgG_1_ specific for all recombinant antigens were similar, except for Tci-MIF-1 and Tci-SAA-1, which were not significantly different to each other, but were significantly lower than the other antigens (Additional file 6). Specific IgG_2_ levels against all vaccine proteins were variable in both breeds; represented in Additional files 7 and 8. In vaccinated CS animals, levels of Tci-ASP-1 were significantly higher than IgG_2_ levels against the other recombinant proteins, except Tci-SAA-1. Levels of IgG_2_ specific for Tci-MIF-1 were lower than most of the other proteins. In general, specific levels of this immunoglobulin isotype against all proteins were variable and individual differences between antigens were recorded. In vaccinated CHB, the individual variability was also high: Specific IgG_2_ levels against Tci-MEP-1 were higher than all the other proteins except Tci-CF-1. On the contrary, specific IgG_2_ levels of Tci-MIF-1 were significantly lower than IgG_2_ levels of all the other vaccine antigens. Detailed significances in IgG_2_ levels specific for the other recombinant proteins in CHB are presented in Additional file 8.

**Table 3:** **Levels of serum immunoglobulins against a subset of recombinant vaccine proteins in Canaria Sheep (CS) and Canaria Hair Breed (CHB) sheep vaccinated with a prototype recombinant sub-unit** ***Teladorsagia circumcincta* vaccine and subsequently challenged with** ***T. circumcincta***

Isotype	Antigen	Group	Mean ± SEM	Cumulative FEC	Worm burden	Worm length	EIU
Correlation							
IgA	Tci-MEP-1	CS Vac	1.656 ± 0.058**	−0.364	−0.650*	−0.497	−0.706*
		CS Con	1.188 ± 0.045**	0.473	0.727*	0.309	0.109
		CHB Vac	1.652 ± 0.071**	−0.073	−0.582	−0.321	−0.358
		CHB Con	1.246 ± 0.085**	0.109	0.399	0.385	−0.021
IgG1	Tci- ASP-1	CS Vac	1.877 ± 0.043**	−0.399	−0.196	−0.392	−0.636*
		CS Con	0.991 ± 0.004**	−0.405	0.223	−0.087	0.219
		CHB Vac	1.683 ± 0.067*	0.155	−0.036	−0.285	−0.358
		CHB Con	1.017 ± 0.013*	0.494	0.126	0.308	0.406
	Tci- SAA-1	CS Vac	2.104 ± 0.086**	−0.552	−0.252	−0.699*	−0.608*
		CS Con	0.995 ± 0.002**	0.018	−0.027	−0.391	−0.600
		CHB Vac	1.478 ± 0.089*	−0.191	−0.118	−0.152	−0.612
		CHB Con	0.999 ± 0.001*	0.182	0.280	−0.119	−0.098
IgG2	Tci- ASP-1	CS Vac	2.004 ± 0.049**	−0.399	−0.329	−0.392	−0.587*
		CS Con	0.998 ± 0.003**	−0.100	−0.073	−0.182	0.509
		CHB Vac	1.701 ± 0.059*	0.164	−0.118	−0.067	−0.164
		CHB Con	1.004 ± 0.001*	0.067	0.112	0.028	−0.021

Antibody levels are expressed as (ODI + 1). Only the antigens for which correlations with parasitology variables were established are shown in the table. Associations are expressed as Spearman’s correlation coefficient. Statistically-significant differences within breeds for a specific isotype and antigen and significant correlations are represented with “*” at *p* < 0.05 and “**” at *p* < 0.01.

Specific IgA levels against Tci-MEP-1 in the serum of vaccinated CS animals were negatively correlated with adult worm burden (*p* < 0.05) and EIU (*p* < 0.05) (Table 3). Levels of specific IgG_1_ against Tci-SAA-1 in the serum of vaccinated CS animals were also negatively correlated with worm length and EIU (*p* < 0.05) and, in this group, levels of specific IgG_1_ and IgG_2_ against Tci-ASP-1 correlated negatively with EIU (*p* < 0.05) (Table 3). No other antigen-specific immunoglobulin isotype in any other vaccinated group was negatively correlated with any parasitological parameter (Additional files 3, 4, 5, 6, 7 and 8).

### Cellular responses

**Figure 1:**
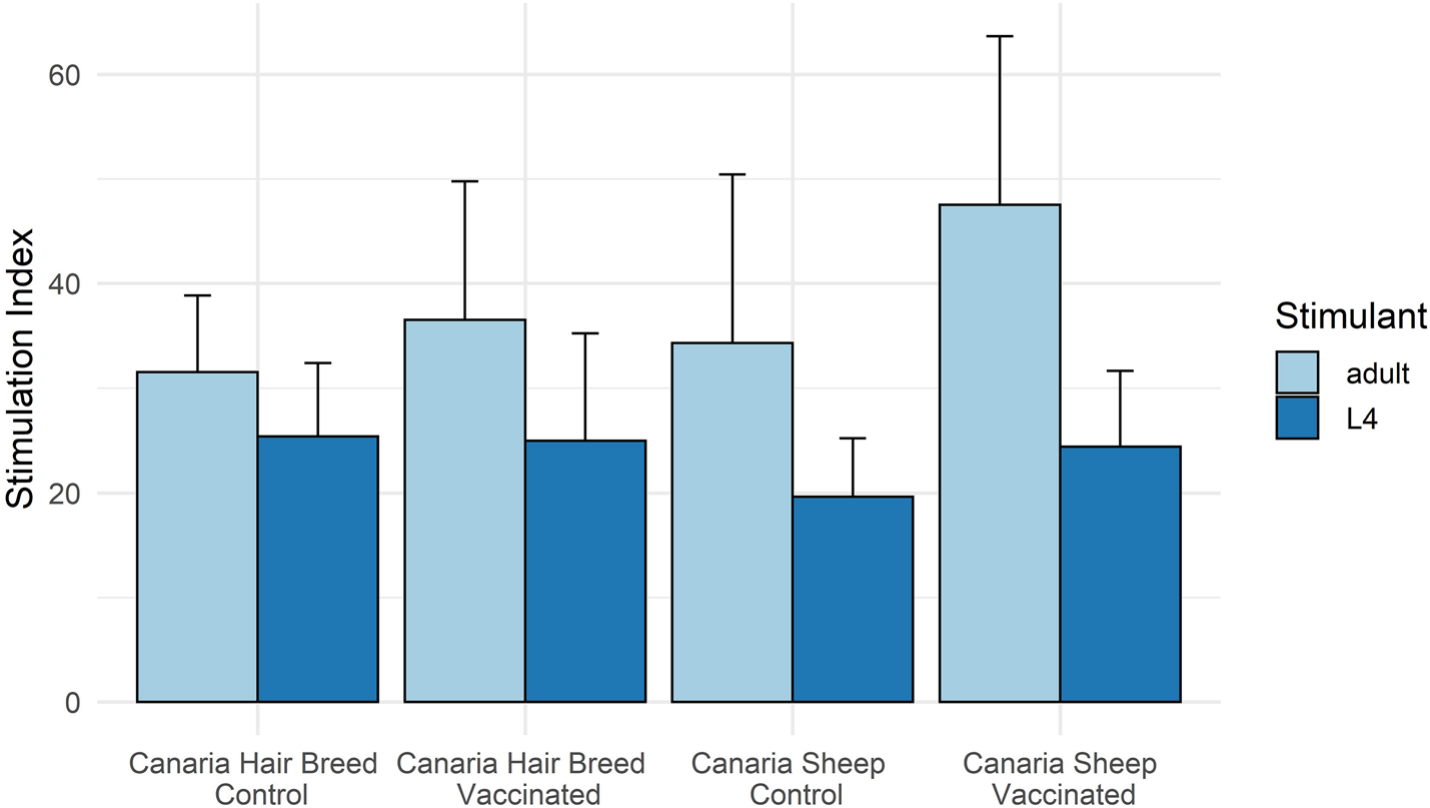
**Antigen specific proliferation in Abomasal Lymph Nodes from Canaria Sheep and Canaria Hair Breed sheep vaccinated with a prototype recombinant sub-unit* Teladorsagia circumcincta*** **vaccine and subsequently challenged with** ***T. circumcincta***. Abomasal Lymph Nodes were collected at post-mortem and lymphocytes stimulated with 5 g/mL of *T. circumcincta* L4 or adult somatic antigen for 5 days. Proliferation was measured by the incorporation of methyl-^3^H thymidine ([^3^H]TdR; 0.5 μCi per well) for the final 18 h of culture. Data are presented as the stimulation index with error bars denoting ± SE.

Abomasal lymph node (ALN) cells from vaccinated animals from both breeds generally showed higher mean proliferative responses to *T. circumcincta* adult somatic antigen compared to L4 somatic antigen (Figure  1). No significant differences in antigen specific proliferation were observed between vaccinated or control animals for either breed, and no vaccine x breed interaction was observed.

**Figure 2:**
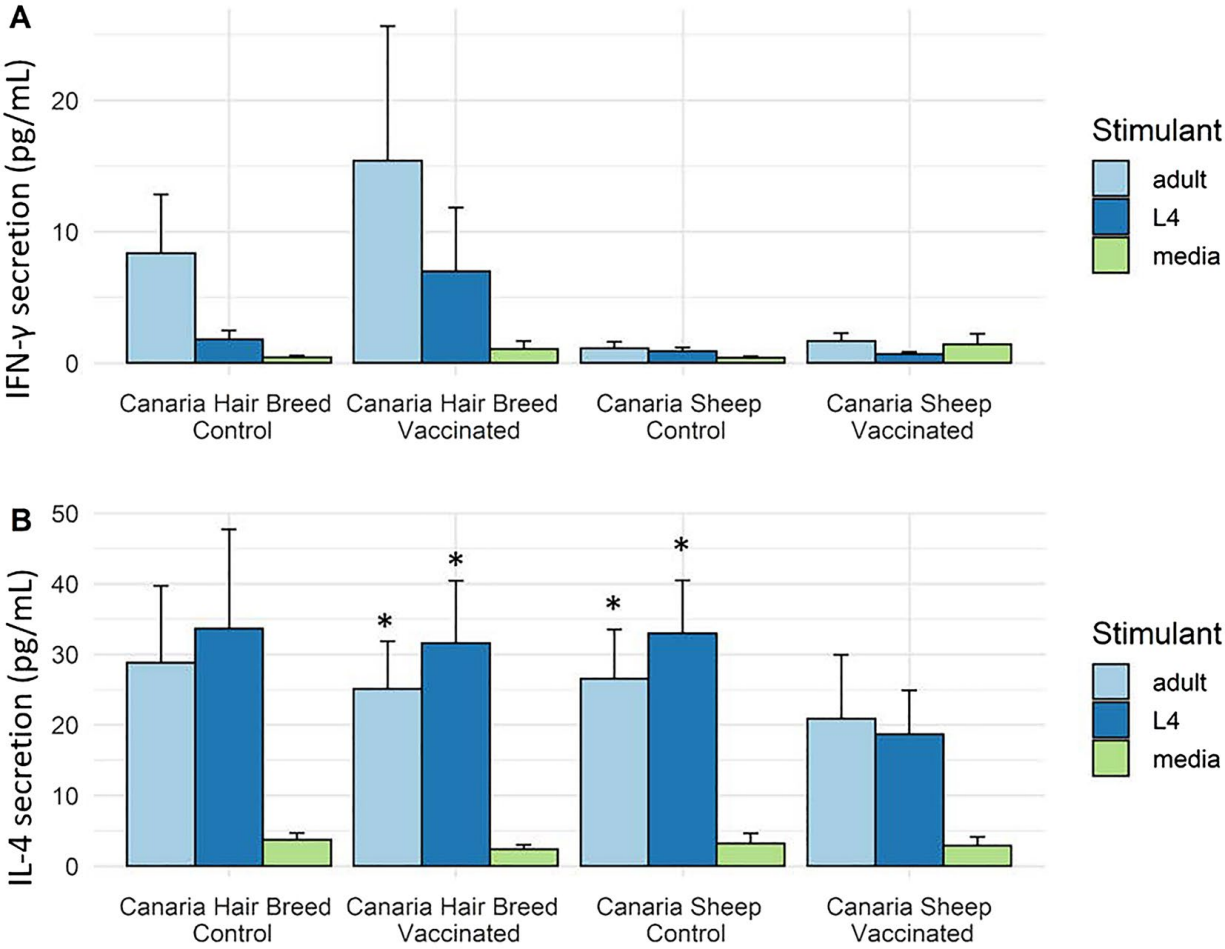
**IFN-γ and IL-4 secretion by abomasal lymph node lymphocytes following stimulation with** ***Teladorsagia circumcincta*** **L4 or adult somatic antigen from Canaria Hair Breed and Canaria Sheep vaccinated with a prototype recombinant sub-unit** ***T. circumcincta* vaccine and subsequently challenged with** ***T. circumcincta***. IFN-γ (**A**) and IL-4 (**B**) secretion was examined in supernatants collected 4 days post-stimulation of abomasal lymph node lymphocytes with 5 g/mL of *T. circumcincta* L4 or adult somatic antigen. Data are expressed as the concentration of the cytokine release in picograms per mL (pg/mL). Results are shown as the mean values with error bars indicating ± SE. “*” denotes statistical significance for *p* < 0.05 when compared to media only secretion.

Supernatant from antigen-stimulated ALN were harvested on day 4 post-stimulation and levels of IFN-γ and IL-4 quantified. Significantly higher levels of IFN-γ were present in ALN cells stimulated with both *T. circumcincta* adult and L4 antigens compare to unstimulated controls (F = 3.72, DF = 2, *p* = 0.027; Figure  2A). There was no evidence of an effect of breed or vaccination status, nor any interactions between vaccination and breed, although mean IFN-γ production was higher in antigen-stimulated ALN cells from CHB sheep. Similarly, IL-4 release was significantly higher in ALN cells stimulated with adult and L4 antigens compared to unstimulated controls (F = 34.98, DF = 2, *p* < 0.001; Figure  2B) but again there was no evidence of a main effect of breed or vaccination status, nor an interaction between vaccination and breed.

Antigen-specific IL-17A release was not detected in any of the ALN cultures (data not shown).

**Figure 3:**
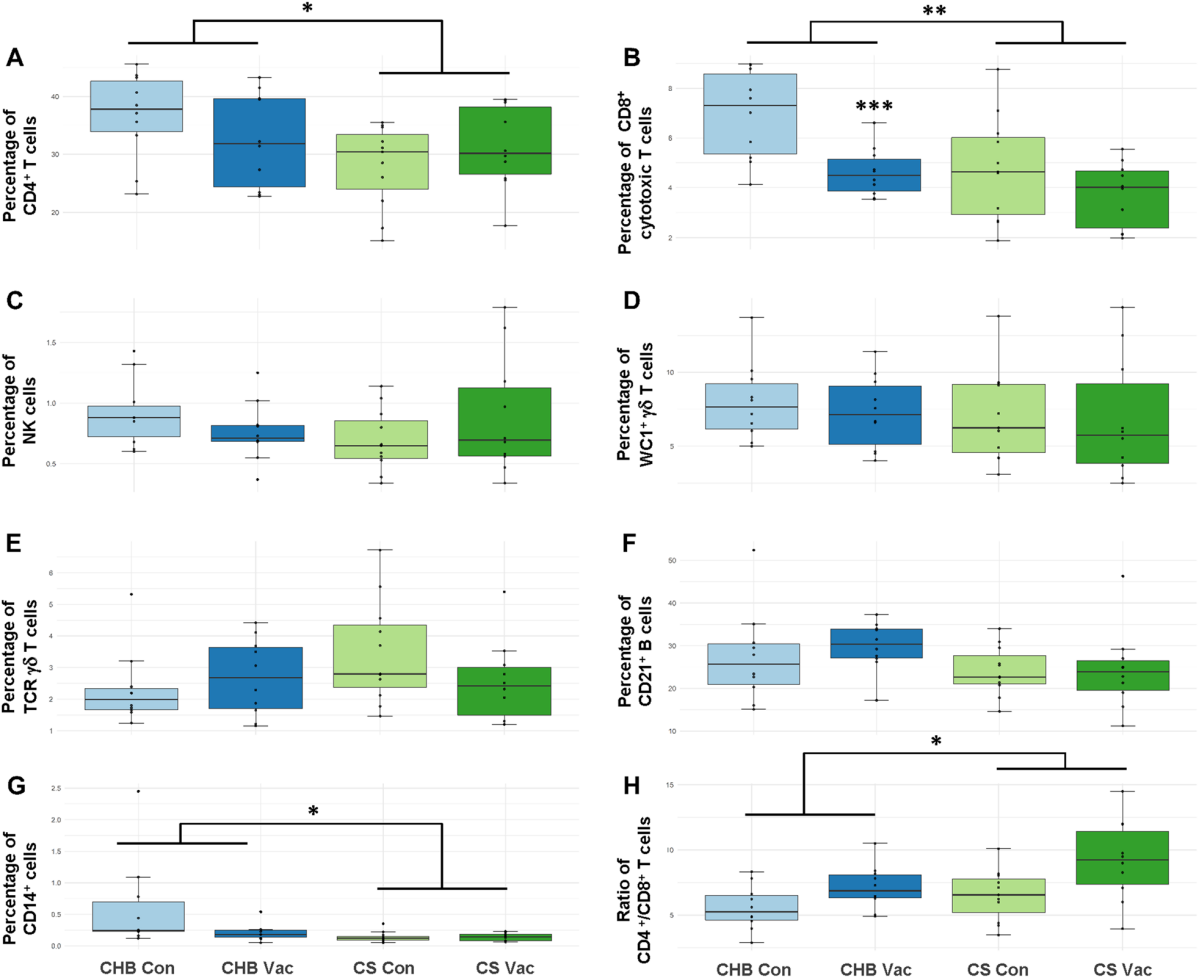
**Phenotypic profile of Canaria Hair Breed (CHB) and Canaria Sheep (CS) abomasal lymph node (ALN) cells following vaccination with a prototype recombinant sub-unit** ***Teladorsagia circumcincta*** **vaccine and subsequent challenge with* T. circumcincta***. The expression of cell surface markers corresponding to main blood cell lineages: T helper CD4^+^ cells, cytotoxic CD8^+^ T cells and γδ^+^ T cells, NK cells, CD21^+^ B cells and CD14^+^ myeloid cells (monocytes and macrophages) were examined by single colour flow cytometry. Cells were gated to eliminate dead cells and doublets. Threshold levels which determined positivity for the selected cell surface markers were set with secondary antibody only. Results are shown as boxplots with IQR and error bars indicating ± SE. “*” indicates statistical significance for *p* < 0.05, “**” *p* < 0.01 and “***” *p* < 0.001. Abbreviations: Vac  Vaccinated, Con  Control (adjuvant-only).

When the phenotype of the ALN cells was investigated by flow cytometry, significant effects of the vaccine and breed were identified. The proportions of CD4^+^ T cells within ALN cell populations were higher in CHB sheep compared to CS sheep (F = 4.42, *p* = 0.042), which appeared largely driven by the control animals (Figure  3A). Similarly, the proportion of CD8^+^ T cells was also significantly higher in CHB sheep (F = 8.72, *p* = 0.005; Figure  3B), and were also higher in controls compared to vaccinated groups (F = 10.74, *p* < 0.002). The proportion of CD14^+^ cells were higher in CHB sheep (F = 5.72, *p* = 0.022; Figure  3G). Finally, the CD4^+^:CD8^+^ ratio was affected by both breed and vaccination, with higher CD4^+^:CD8^+^ ratios observed in the CS breed (H = 4.52, *p* = 0.04) and in vaccinated animals (F = 9.77, *p* = 0.003) (Figure  3H).

**Figure 4:**
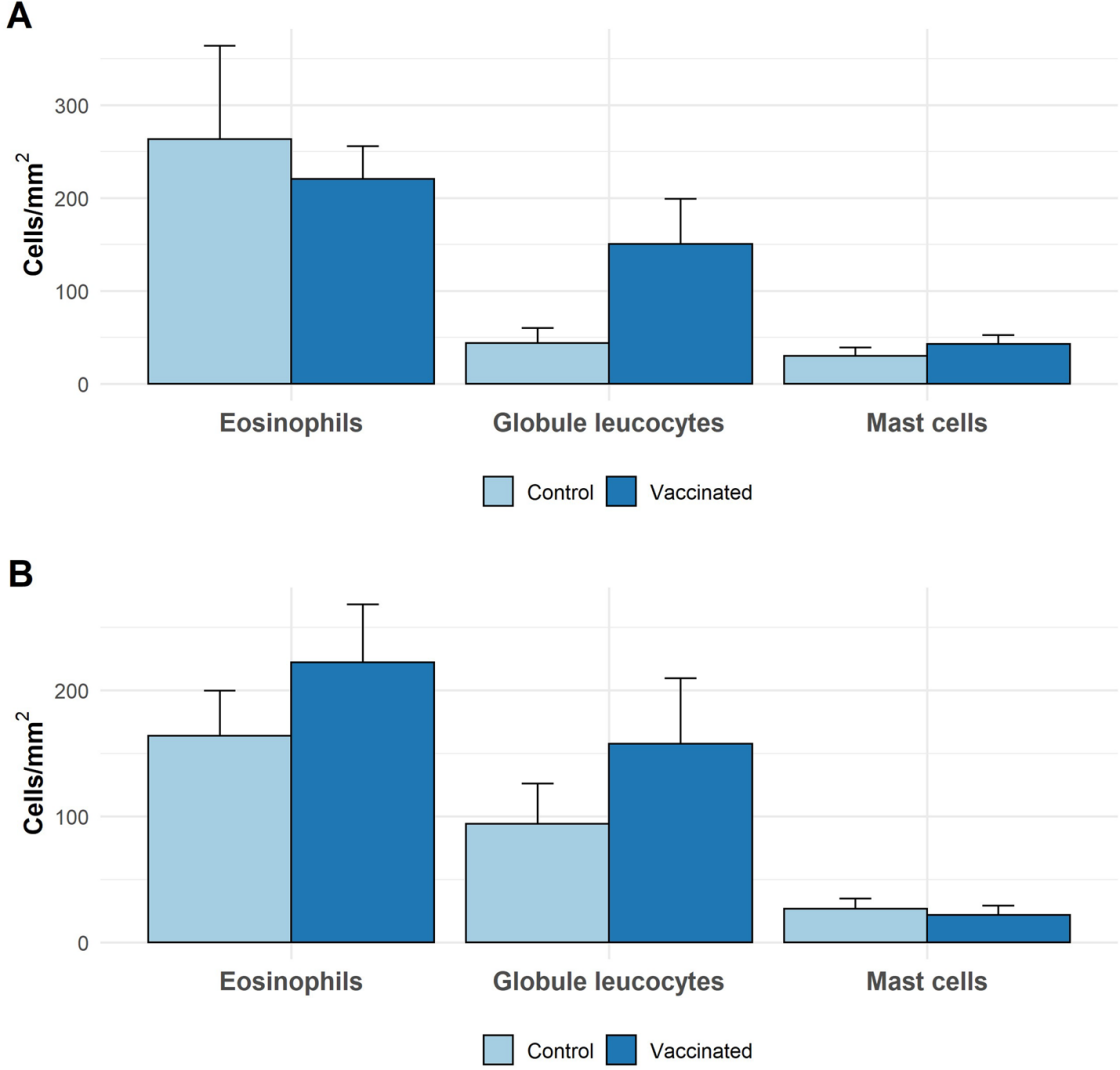
**Eosinophils, globule leucocytes and mast cells in the abomasal wall in Canaria Sheep (A) and Canaria Hair Breed (B) following vaccination with a prototype recombinant sub-unit* Teladorsagia circumcincta* vaccine and subsequent** ***T. circumcincta*** challenge. Values shown as means (cells/mm^2^) ± SEM in abomasal tissue obtained at post-mortem following vaccination and parasite challenge.

**Figure 5:**
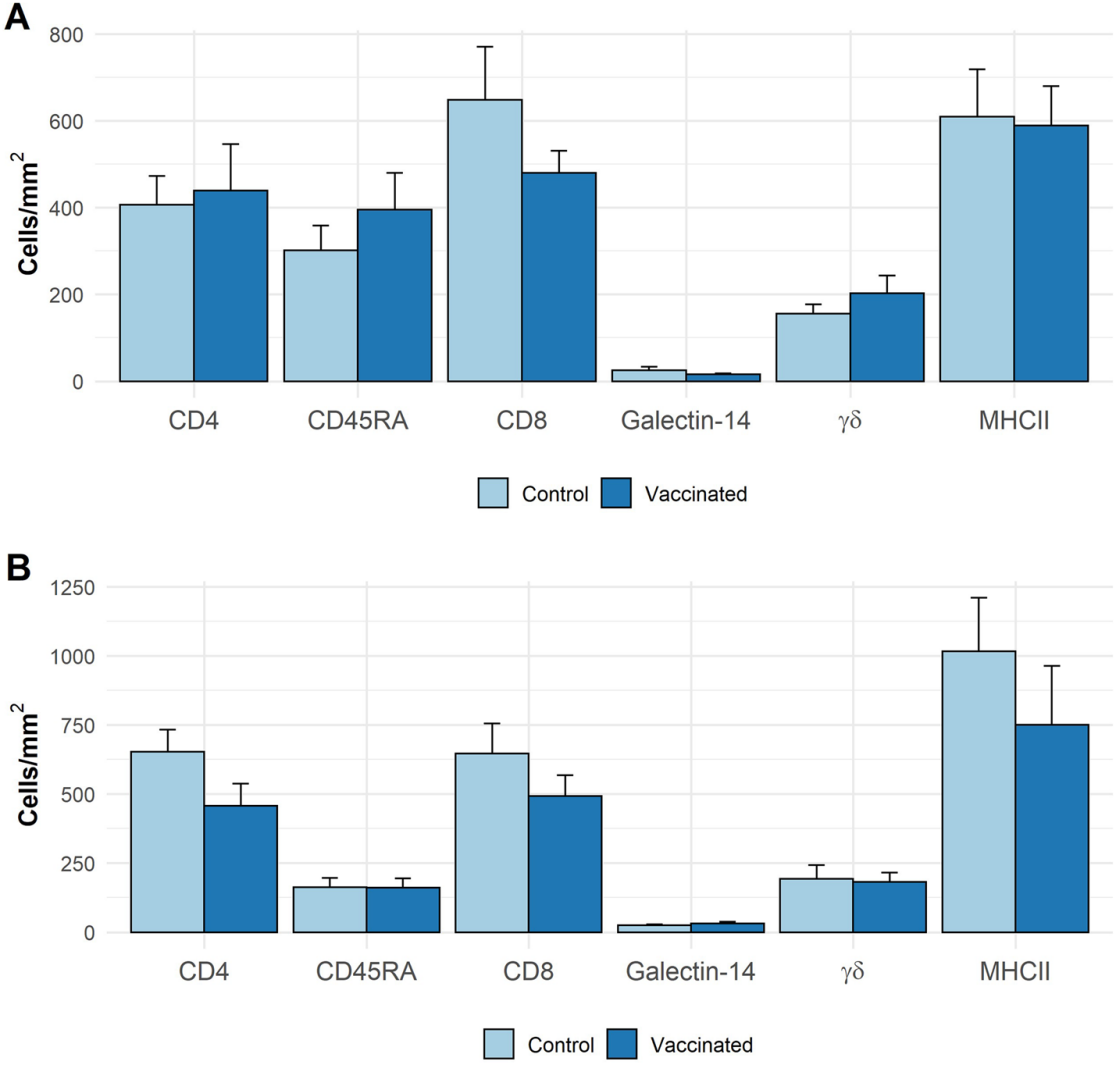
**CD4, CD45RA, CD8, galectin-14, γδ and MHCII positive cells in the abomasal mucosa in Canaria Sheep (A) and Canaria Hair Breed (B) following vaccination with a prototype recombinant sub-unit * Teladorsagia circumcincta * vaccine and subsequent* T. circumcincta* challenge**. Values shown are means (cells/mm^2^) ± SEM in abomasal tissue obtained at post-mortem following vaccination and parasite challenge.

No differences in mean values of eosinophils, mast cell, GLs, CD4^+^, CD8^+^, CD45RA^+^, MHCII^+^, γδ^+^ and galectin-14^+^ populations in the abomasal mucosa were observed between vaccinated and non-vaccinated animals of either breed (Figures 4 and 5). Globule leukocyte (GL) levels showed negative correlations with several parasitological variables analysed in all groups, but particularly in vaccinated CS animals in which GL levels were negatively associated with all of the parasitology measures recorded (Table 4). No other cell population studied in vaccinated groups showed a negative association with parasitology in any group (Additional files 9 and 10).

**Table 4:** **Relationship between globule leukocytes and parasitological variables in Canaria Sheep (CS) and Canaria Hair Breed (CHB) sheep vaccinated with a prototype recombinant sub-unit** ***Teladorsagia circumcincta* vaccine and subsequently challenged with** ***T. circumcincta***

Group	Cumulative FEC	Worm burden	Worm length	EIU
CS Vac	−0.811**	−0.608*	−0.776**	−0.734**
CS Con	−0.409	−0.182	−0.764**	−0.545
CHB Vac	−0.545	−0.555	−0.309	−0.285
CHB Con	−0.664*	−0.630*	−0.340	−0.364

Significant correlations are represented with “*” at *p* < 0.05 and “**” at *p* < 0.01.

## Discussion

Naturally or experimentally-induced protection against *T. circumcincta* following infection is a complex phenomenon in which several immunoglobulins such as IgA, IgE, IgG, cells such as eosinophils, mast cells, globule leukocytes (GL), CD4^+^, plasmatic cells and other factors, like IL-4 and/or 5 or galectins are implicated. Although the main “actors” of this response are well-known, there is great variability in the phenotype of immunity. Part of this variability is a consequence of host factors such as age, breed, genetic resistance, but also parasite factors such as stage of worm development and level of burden [3].

The fact that lambs naturally acquire protective immunity against GIN after continual field-infection, underpinned the concept of developing a vaccine against these types of pathogens (recently reviewed in Britton et al. [16]). But, because of the complexity of the protective mechanisms involved in natural immunity, it is a challenge to recreate these with a recombinant subunit vaccine. Certainly, several promising antigens have been identified, but most of them share similar limitations: (1) it may not be possible to produce them as effective recombinant antigens; (2) there is individual variability in protection conferred by vaccines [9]. Recently, a recombinant prototype against *T. circumcincta* was identified which successfully protected lambs and periparturient ewes [7, 8]. However, individual variability in responses was observed. Furthermore, a reduction in the number of immunogens would be desirable for decreasing vaccine production cost. This type of reductionist strategy needs to be led by an understanding of vaccine-induced immunity and, if possible, immunological correlates of protection.

In the work presented here, the reduction of worm length and EIU in vaccinated CS groups was negatively correlated with levels of IgA and IgG_2_ specific for extracts of L4 stage *T. circumcincta*. The role of IgA in protection of sheep against *T. circumcincta* has been proposed in previous studies of natural immunity, where it has been related to regulating worm growth targeting the L4 stage of this parasite [1, 17]. Several studies have associated IgA, IgG_1_ and IgE with natural resistance against several GIN in sheep [18, 19]. IgG_2_ has been also associated with natural protection against larval stages of *Trichostrongylus colubriformis* in genetically resistant sheep [20]. Interestingly, high levels of total IgG against L4 and excretory/secretory (ES) antigens of L4 were observed in Texel crossbred after vaccination [8]. Production of specific IgA against L4 *T. circumcincta* was also increased in vaccinated CHB. All the data suggest that a humoral response against L4 antigens is a consistent response possibly induced by this vaccine, which is logical as many of the antigens were selected based on their reactivity with IgA in the abomasal lymph or mucus from immune sheep [8], although abomasal lymph node cells were more strongly stimulated by adult *T. circumcincta* antigens than by L4 antigens, potentially due to immunomodulatory and/or suppressive molecules within L4 [21].

Globule leukocytes are considered to be activated mast cells [22]. Correlation studies also suggest a role for GL in the protection conferred by the vaccine in both breeds, and levels of this cell type were also negatively correlated with some elements of the parasitology in control groups. IL-4 is a key cytokine in mast cell maturation and, in agreement with histological data, it was also identified at high levels in ALN cells in all groups, independent of breed or vaccination status, following exposure to L4 and adult antigens. Globule leukocytes have been previously associated with resistance against GIN in animals naturally resistant to *T. circumcincta* [23, 24], generating a rapid larval rejection [25]. The data obtained here suggest that the vaccine confers protection in these breeds partly through improving mostly “natural mechanisms” of protection.

This vaccine prototype has now protected Texel crossbred lambs in five different trials [9] and, in this assay, also CS lambs were significantly protected. In CHB, all parasitological parameters were reduced in vaccinated group, although the reductions were not statistically significant compared to the control group. With the current data, it is not possible to confirm if the CHB lambs did not respond to the vaccine or if this breed is so naturally resistant to *T. circumcincta* that the vaccine could not add extra protection. In fact, the adjuvant-only control CHB group harboured fewer and shorter worms and excreted fewer eggs than either vaccinated or control CS lambs. Cellular and humoral response studied in this trial were, however, very similar between the breeds, with ALN cells from both breeds releasing IFN-γ and IL-4 following stimulation with larval and adult worm antigens. This indicates that in both breeds, infection with *T. circumcincta* induced a mixed T helper type 1 (Th1) / Th2 immune response, with possibly higher levels of IFN-γ produced by CHB lambs. While Th1 immunity, characterised by IFN-γ, has been linked with susceptibility to gastrointestinal nematodes [26], other studies indicate that Th1 immunity can be linked with resistance to *H. contortus*, for example, Caribbean hair sheep show higher IFN-γ production than susceptible sheep at 4 weeks post-infection [27]. More recently, a study in Creole goats has shown that resistance to *H. contortus* is associated with an upregulation of Th1 immune associated genes by 5 weeks post-infection, but that infection induces similar Th2 immune responses in resistant and susceptible goats [28]. These results provide growing evidence that a mixed Th1/Th2 immune response may be optimal for protection against ruminant GIN.

The main differences in cellular immune phenotypes between the two breeds were observed in ALN cell populations at post-mortem, whereby the proportion of CD4^+^ and CD8^+^ T cells within the ALN cells were higher in CHB lambs, and in particular the controls. CD4^+^:CD8^+^ ratios were also higher in vaccinated lambs of both breeds. These results suggest that the CHB induces stronger local adaptive immune responses, reflected by higher levels of recruitment to and expansion of T cells within the ALN. It also indicates that vaccination via the systemic route has had some influence on the balance of CD4^+^ and CD8^+^ T cells within the LN draining the site of infection, with a greater proportion of CD4^+^ T cells possibly reflecting reactivation of memory CD4^+^ T cells primed by the vaccine. While induction of mucosal immune responses via the systemic route is generally ineffective, this has previously been shown for *O. ostertagi*, a GIN of cattle closely related to *T. circumcincta*, where intra-muscular immunisation of cattle with native ASP-1 and Quil A induced antigen-specific proliferation of natural killer (NK) cells in the abomasal mucosa [29]. Interestingly, this NK response was lost when the antigen was formulated with Al(OH)_3_, suggesting that the Quil A adjuvant, which was also used in this study, may be critical for systemically induced priming of the abomasal immune response.

As pointed out above, it would be desirable to reduce the number of recombinant proteins for commercial purposes [9]. Previous trials performed with Texel crossbred lambs have indicated Tci-APY-1 and Tci-MEP-1 as the most promising candidates for inclusion in a simplified vaccine for *T. circumcincta* [9]. Immunoglobulin levels and/or antibody:antigen avidity, relationship with parasitological parameters and recognition by adjuvant-only control lambs as they developed immunity following challenge, were the criteria for selecting these two proteins from the whole cocktail. The administration of Tci-MEP-1 and a mutated version of Tci-APY-1 performed as well as the whole vaccine prototype in Texel crossbred [9]. Tci-MEP-1 may also have a protective role in CS and CHB sheep in the trial presented here because in both breeds, specific serum IgA against this protein was negatively associated with parasitological parameters. The IgA response to Tci-APY-1 also showed negative associations with worm burden in CHB sheep. It seems that these two proteins could have a role in protection conferred by this prototype.

However, data obtained with CS and CHB lambs showed some slightly different responses. In CS, following similar criteria, Tci-SAA-1 and Tci-ASP-1 would also be good candidates, because IgG_1_ against both proteins and IgG_2_ to Tci-ASP-1 was also negatively correlated with EIU. Interestingly, in Texel cross bred lambs, in which no differences in worm length were detected [7], these associations were not observed. This could mean breed differences in vaccine protective mechanisms. Future studies must clarify why this response was apparently breed-dependent as it appears that the host breed is important in this response. Considering the humoral response, therefore, it is not clear which recombinant protein(s) should be selected to ensure protective immunity across several breeds, although Tci-MEP-1, Tci-APY-1 are promising candidates that have also been successfully tested [9].

Some of these proteins may not induce a strong humoral response but could trigger a critical cellular response. In fact, some successful vaccine prototypes against related GIN develop their protective mechanisms through cellular responses [30–32]. Noticeably, GLs were negatively associated with protection in both vaccinated breeds in this trial.

In conclusion, this recombinant vaccine protected six-month-old CS breed lambs and did not significantly reduce parasitological parameters in CHB sheep. Globule leukocytes and IgA and IgG_2_ to L4 *T. circumcincta* antigens may be key mechanisms in protection conferred by the vaccine in the CS breed. Improving these responses at vaccination could reduce individual variability and enhance the response. Future studies considering sequential analysis of local immune response and/or depleting particular cell populations, including serially diluted samples at different times of infection for refining antibodies measurement accuracy. All these studies would be desirable for improving this vaccine prototype.

## Supplementary Information

**Additional file 1. Antibody clones used for flow cytometry analysis and immunohistochemistry.** “*” Antibody clone used for flow cytometry assays. “**” Antibody clone used for IHQ.

**Additional file 2. Examples of abomasal mucosa sections from Canaria Hair Breed and Canaria Sheep experimentally infected with * Teladorsagia circumcincta* showing positive cells (x400).** Haematoxylin and eosin staining for globule leukocyte in the upper layer (**A**) and eosinophils in the basal layer (**B**), both indicated with arrows. Immunohistochemical staining with CD4+ antibody (SBU T4 pool 44.38 + 44.97), showing CD4+ cells (arrows) in the apical (**C**) and basal area (**D**).

**Additional file 3.** **IgA against vaccine proteins and their correlation with parasitology in Canaria Sheep lambs.** Associations are expressed as Spearman’s correlation coefficient. Same letters mean no significant differences between groups. Significant correlations are represented with “*” at p < 0.05 and “**” at p < 0.01.

**Additional file 4. IgA against vaccine proteins and their correlation with parasitology in Canaria Hair Breed lambs. ** Associations are expressed as Spearman’s correlation coefficient. Same letters mean no significant differences between groups. Significant correlations are represented with “*” at p < 0.05 and “**” at p < 0.01.

**Additional file 5. IgG1 against vaccine proteins and their correlation with parasitology in Canaria Sheep lambs.** Associations are expressed as Spearman’s correlation coefficient. Same letters mean no significant differences between groups. Significant correlations are represented with “*” at p < 0.05 and “**” at p < 0.01.

**Additional file 6. IgG1 against vaccine proteins and their correlation with parasitology in Canaria Hair Breed lambs.** Associations are expressed as Spearman’s correlation coefficient. Same letters mean no significant differences between groups. Significant correlations are represented with “**” at p < 0.01.

**Additional file 7. IgG2 against vaccine proteins and their correlation with parasitology in Canaria Sheep lambs.** Associations are expressed as Spearman’s correlation coefficient. Same letters mean no significant differences between groups. Significant correlations are represented with “*” at p < 0.05 and “**” at p < 0.01.

**Additional file 8. IgG2 against vaccine proteins and their correlation with parasitology in Canaria Hair Breed lambs.** Associations are expressed as Spearman’s correlation coefficient. Same letters mean no significant differences between groups. Significant correlations are represented with “*” at p < 0.05 and “**” at p < 0.01.

**Additional file 9. Correlations between cells and parasitological variables in Canaria Sheep lambs.** Associations are expressed as Spearman’s correlation coefficient. Significant correlations are represented with “*” at p < 0.05.

**Additional file 10. Correlations between cells and parasitological variables in Canaria Hair Breed lambs.** Associations are expressed as Spearman’s correlation coefficient.

**Additional file 11: Interleukin-17A secretion by abomasal lymph node lymphocytes following stimulation with** ***Teladorsagia circumcincta*** **L4 or adult somatic antigen from Canaria Hair Breed and Canaria Sheep vaccinated with a prototype recombinant sub-unit** ***T. circumcincta* vaccine and subsequently challenged with *T. circumcincta***. IL-17A secretion was examined in supernatants collected 4 days post-stimulation of abomasal lymph node lymphocytes with 5 µg/mL of *T. circumcincta* L4 or adult somatic antigen. In general, antigen-specific IL-17A release was not detected in any of the ALN cultures.* N/D* IL-17A were not detected,* N/S* no sampled availability.

## Abbreviations

γδTCR: Gamma Delta T Cell Receptor
ALN: Abomasal Lymph Node
BSA: Bovine Serum Albumin
CHB: Canaria Hair Breed
CS: Canaria Sheep
ConA: Concanavalin A
DMSO: Dimethyl Sulfoxide
EIU: Eggs in utero
ES: Excretory/Secretory
FACS: Fluorescence-Activated Cell Sorting
FBS: Fetal Bovine Serum
FCS: Fetal Calf Serum
FEC: Faecal egg counts
GENLIN: Generalised Linear Models
GIN: Gastrointestinal nematodes
GL: Globule Leukocyte
GLM: General Linear Models
HBSS: Hank’s Balanced Salt Solution
*H. contortus*: *Haemonchus contortus*
Ig: Immunoglobulin
IL-4: Interleukin 4
IL-17A: Interleukin 17A
IFN-γ: Interferon gamma
L3: Third stage larval
L4: Fourth stage larval
LN: Lymph Node
LSA: Lymphocyte Stimulation Assays
LSD: Least Significance Difference
MHCII: Major Histocompatibility Complex II
M: Molar
NK: Natural Killer cells
OD: Optical Density
ODI: Optical Density Index
*O. ostertagi*: *Ostertagia ostertagi*
OPD: O-phenylene-diamine dihydrochloride
PBS: Phosphate buffered saline
RPMI: Roswell Park Memorial Institute medium
SEM: Standard error of the mean
SI: Stimulation Index
TBST: Tris Buffered Saline containing 0.1% Tween®20
*T. circumcincta*: *Teladorsagia circumcincta*
Tci-APY-1: Calcium-dependent apyrase-1
Tci-ASP-1: Activation-associated secretory protein-1
Tci-CF-1: Cathepsin F-1
Tci-ES20: 20 KDa protein of unknown function
Tci-MEP-1: Astacin-like metalloproteinase-1
Tci-MIF-1: Macrophage migration inhibitory factor-1
Tci-SAA-1: An homologue of a protective antigen from *Ancylostoma caninum*
Tci-TGH-2: TGF homologue
Th1: T helper type 1
Th2: T helper type 2
TWM: Transport Wash Media
TMB: 3,3', 5, 5' – Tetramethylbenzidine

## References

[1] Stear MJ, Bishop SC, Doligalska M, Duncan JL, Holmes PH, Irvine J, McCririe L, McKellar QA, Sinski E, Murray M (1995). Regulation of egg production, worm burden, worm length and worm fecundity by host responses in sheep infected with *Ostertagia circumcincta*. Parasite Immunol.

[2] Stear MJ, Bishop SC (1999). The curvilinear relationship between worm length and fecundity of *Teladorsagia circumcincta*. Int J Parasitol.

[3] McRae KM, Stear MJ, Good B, Keane OM (2015). The host immune response to gastrointestinal nematode infection in sheep. Parasite Immunol.

[4] Smith WD, Jackson F, Jackson E, Williams J (1985). Age immunity to *Ostertagia circumcincta*: Comparison of the local immune responses of 4 ½ - and 10-month-old lambs. J Comp Pathol.

[5] Henderson NG, Stear MJ (2006). Eosinophil and IgA responses in sheep infected with *Teladorsagia circumcincta*. Vet Immunol Immunopathol.

[6] Vercruysse J, Charlier J, Van Dik J, Morgan ER, Geary T, von Samson-Himmelstjerna G, Claerebout E (2018). Control of helminth ruminant infections by 2030. Parasitol.

[7] Nisbet AJ, McNeilly TN, Greer AW, Bartley Y, Oliver EM, Smith S, Palarea-Albaladejo J, Matthews JB (2016). Protection of ewes against *Teladorsagia circumcincta* infection in the periparturient period by vaccination with recombinant antigens. Vet Parasitol.

[8] Nisbet AJ, McNeilly TN, Wildblood LA, Morrison AA, Bartley DJ, Bartley Y, Longhi C, McKendrick IJ, Palarea-Albaladejo J, Matthews JB (2013). Successful immunization against a parasitic nematode by vaccination with recombinant proteins. Vaccine.

[9] Nisbet AJ, McNeilly TN, Price DRG, Oliver EM, Bartley Y, Mitchell M, Palarea-Albaladejo J, Matthews JB (2019). The rational simplification of a recombinant cocktail vaccine to control the parasitic nematode *Teladorsagia circumcincta*. Int J Parasitol.

[10] Matthews JB, Geldhof P, Tzelos T, Claerebout E (2016). Progress in the development of subunit vaccines for gastrointestinal nematodes of ruminants. Parasite Immunol.

[11] González JF, Hernández JN, Machín C, Pérez-Hernández T, Wright HW, Corripio-Miyar Y, Price D, Matthews JB, McNeilly TN, Nisbet AJ (2019). Impacts of breed type and vaccination on *Teladorsagia circumcincta* infection in native sheep in Gran Canaria. Vet Res.

[12] MAFF (1989) Manual of Veterinary Parasitological Laboratory Diagnostic Techniques, third ed. Ministry of Agriculture, Fisheries and Food, London

[13] Smith SK, Nisbet AJ, Meikle L, Inglis N, Sales J, Beynon RJ, Matthews JB (2009). Proteomic analysis of excretory/secretory products released by *Teladorsagia circumcincta* larvae early post-infection. Parasite Immunol.

[14] Strain SA, Stear MJ (2001). The influence of protein supplementation on the immune response to *Haemonchus contortus*. Parasite Immunol.

[15] Hernández JN, Meeusen E, Stear M, Rodríguez F, Piedrafita D, González JF (2017). Modulation of *Haemonchus contortus* infection by depletion of γδ^+^ T cells in parasite resistant Canaria Hair Breed sheep. Vet Parasitol.

[16] Britton C, Emery D, McNeilly TN, Nisbet AJ, Stear MJ (2020). The potential for vaccines against scour worms of small ruminants. Int J Parasitol.

[17] Stear MJ, Boag B, Cattadori I, Murphy L (2009). Genetic variation in resistance to mixed, predominantly *Teladorsagia circumcincta* nematode infections of sheep: from heritabilities to gene identification. Parasite Immunol.

[18] Stear MJ, Bairden K, Innocent G, Mitchell S, Strain S, Bishop S (2004). The relationship between IgA activity against 4th stage larvae and density dependent effects on the number of 4th stage larvae of *Teladorsagia circumcincta* in naturally infected sheep. Parasitol.

[19] Shaw RJ, Morris CA, Green RS, Wheeler M, Bisset SA, Vlassoff A, Douch PGC (1999). Genetic and phenotypic relationships among *Trichostrongylus colubriformis*-specific immunoglobulin E, anti-*Trichostrongylus colubriformis* antibody, immunoglobulin G1, faecal egg count and body weight traits in grazing Romney lambs. Livest Prod Sci.

[20] Pernthaner A, Cole SA, Morrison L, Green R, Shaw RJ, Hein WR (2006). Cytokine and antibody subclass responses in the intestinal lymph of sheep during repeated experimental infections with the nematode parasite *Trichostrongylus colubriformis*. Vet Immunol Immunopathol.

[21] McNeilly TN, Rocchi M, Bartley Y, Brown JK, Frew D, Longhi C, McLean L, McIntyre J, Nisbet AJ, Wattegedera S, Huntley JF, Matthews JB (2013). Suppression of ovine lymphocyte activation by *Teladorsagia circumcincta* larval excretory-secretory products. Vet Res.

[22] Nisbet AJ, Meeusen EN, González JF, Piedrafita DM (2016). Immunity to *Haemonchus contortus* and vaccine development. Adv Parasitol.

[23] Gruner L, Cortet J, Sauvé C, Hoste H (2004). Regulation of *Teladorsagia circumcincta* and *Trichostrongylus colubriformis* worm populations by grazing sheep with differing resistance status. Vet Res.

[24] Albuquerque ACA, Bassetto CC, Almeida FA, Almeida FA, Hildersley KA, McNeilly TN, Britton C, Amarante AFT (2019). Differences in immune responses to *Haemonchus contortus* infection in the susceptible Ile de France and the resistant Santa Ines sheep under different anthelmintic treatments regimens. Vet Res.

[25] Balic A, Bowles VM, Meeusen ENT (2000). The immunobiology of gastrointestinal nematode infections in ruminants. Adv Parasitol.

[26] Hassan M, Hanrahan JP, Good B, Mulcahy G, Sweeney T (2011). A differential interplay between the expression of Th1/Th2/Treg related cytokine genes in *Teladorsagia circumcincta* infected DRB1*1101 carrier lambs. Vet Res.

[27] MacKinnon KM, Burton JL, Zajac AM, Notter DR (2009). Microarray analysis reveals difference in gene expression profiles of hair and wool sheep infected with *Haemonchus contortus*. Vet Immunol Immunopathol.

[28] Aboshady HM, Mandonnet N, Félicité Y, Hira J, Fourcot A, Barbier C, Johansson AM, Jonas E, Bambou JC (2020). Dynamic transcriptomic changes of goat abomasal mucosa in response to *Haemonchus contortus* infection. Vet Res.

[29] González-Hernández A, Van Coppernolle S, Borloo J, Van Meulder F, Paerewijck O, Peelaers I, Leclercq G, Claerebout E, Geldhof P (2016). Host protective ASP-based vaccine against the parasitic nematode *Ostertagia ostertagi* triggers NK cell activation and mixed IgG1-IgG2 response. Sci Rep.

[30] Karanu FN, McGuire TC, Davis WC, Besser TE, Jasmer DP (1997). CD4+ T lymphocytes contribute to protective immunity induced in sheep and goats by *Haemonchus contortus* gut antigens. Parasite Immunol.

[31] González-Sánchez VM, Cuquerella M, Alunda JM (2018). Vaccination of lambs against Haemonchus contortus with the recombinant rHc23 Effect of adjuvant and antigen dose. PLoS One.

[32] González-Sánchez ME, Ndombasi-Bokuy M, Cuquerella M, Alunda JM (2019). Immunization with recombinant rHc23 partially protects lambs against trickle infections by *Haemonchus contortus*. BMC Vet Res.

[33] Maddox JF, Mackay CR, Brandon MR (1985). Surface antigens, SBU-T4 and SBU-T8 of sheep lymphocyte subsets defined by monoclonal antibodies. Immunology.

[34] Mackay CR, Beya MF, Matzinger P (1989). y/6 T cells express a unique surface molecule appearing late during thymic development. Eur J Immunol.

[35] Ballingall KT, Dutia BM, Hopkins J, Wright H (1995). Analysis of the fine specificities of sheep major histocompatibility complex class II-specific monoclonal antibodies using mouse l-cell transfectants. Anim Genet.

