# Supplementary material for: Cellular and humoral immune responses associated with protection in sheep vaccinated against Teladorsagia circumcincta
Source: Vet Res. 2021 Jun 16;52:89. doi: 10.1186/s13567-021-00960-8 (PMC8207578; doi:10.1186/s13567-021-00960-8)
Supplement: Supplementary file 1 — Additional file 1. Antibody clones used for flow cytometry analysis and immunohistochemistry. “*” Antibody clone used for flow cytometry assays. “**” Antibody clone used for IHQ. [file 13567_2021_960_MOESM1_ESM.docx]

| **Antigen** | **Marker for** | **Antibody clone** | **Isotype** | **Reference** |
| --- | --- | --- | --- | --- |
| CD4 | T helper cells | SBU T4 pool 44.38+44.97 | IgG_2_a+IgG_1_ | [33] |
| CD8 | Cytotoxic T cells | SBU T8 pool 33-65 | IgG_2_a | [33] |
| NKp46 | Natural killer cells | EC1.1 | IgG_1_ | BioRad |
| WC1 | WC1^+^ γδ T cells | SBU T19 19.19* | IgG_2_ | [34] |
| γδTCR | γδ T | 86D** | IgG_1_ | [34] |
| CD21 | B cells | CC21 | IgG_1_ | BioRad |
| CD14 | Myeloid cells | TUK4 | IgG_1_ | BioRad |
| MHCII | Antigen presentation | 28.1 | IgG_1_ | [35] |
| CD45RA | Naive T cells | SBU-P220 | IgG_1_ | [15] |
| Galectin-14 | Released by eosinophils | EL 1-2 | IgG_1_ | [15] |
