# Supplementary figures and images for: Cellular and humoral immune responses associated with protection in sheep vaccinated against Teladorsagia circumcincta
Source: Vet Res. 2021 Jun 16;52:89. doi: 10.1186/s13567-021-00960-8 (PMC8207578; doi:10.1186/s13567-021-00960-8)

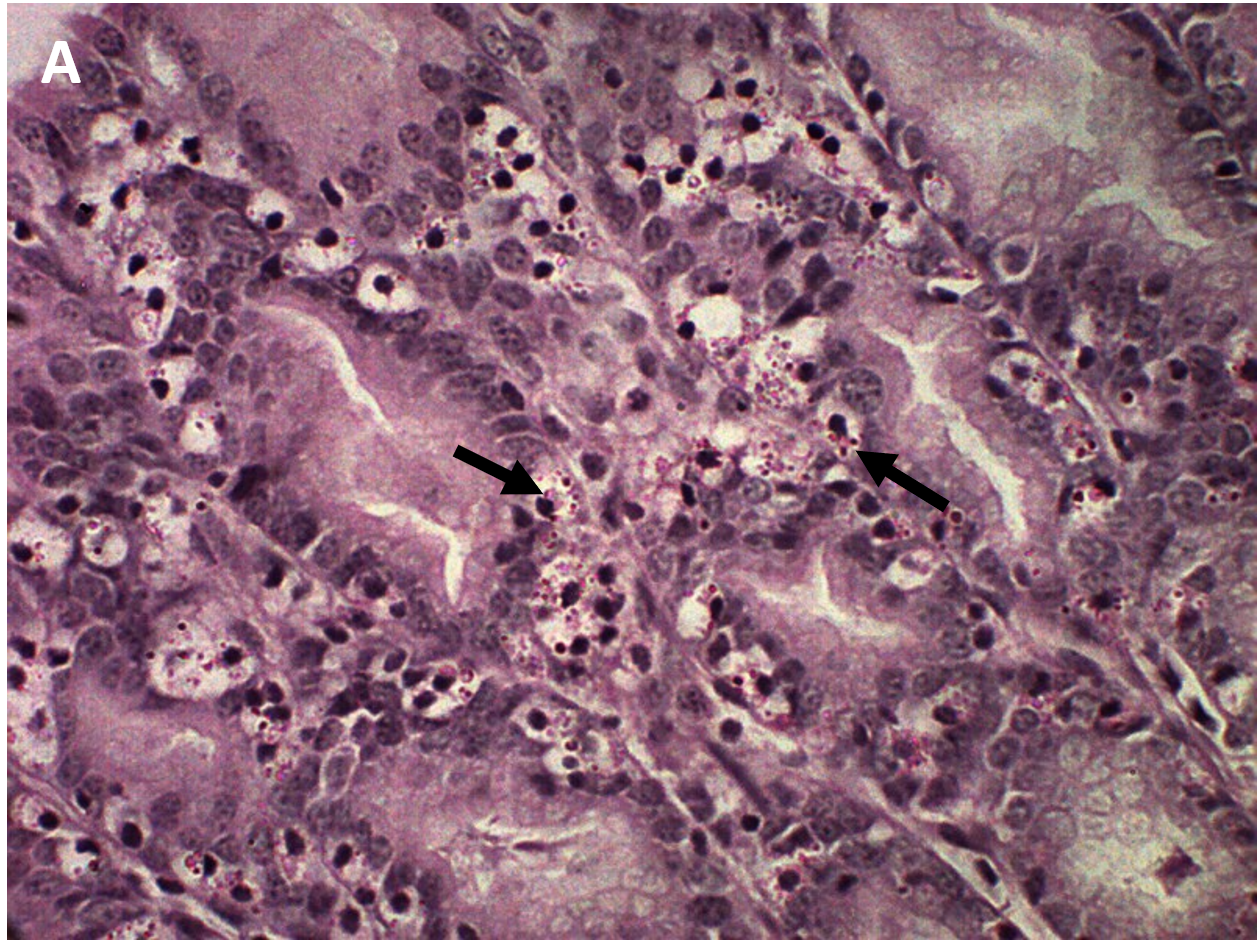


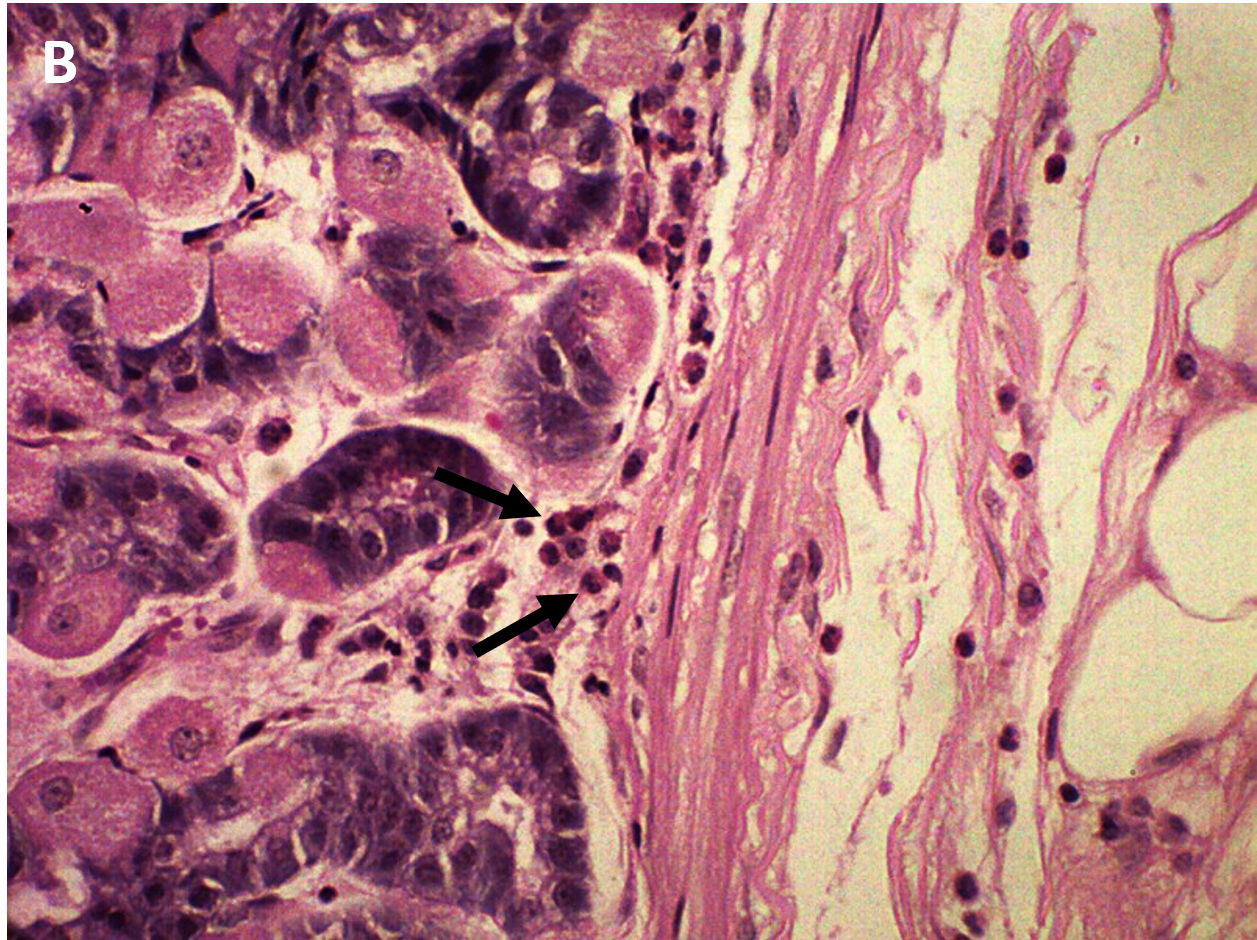


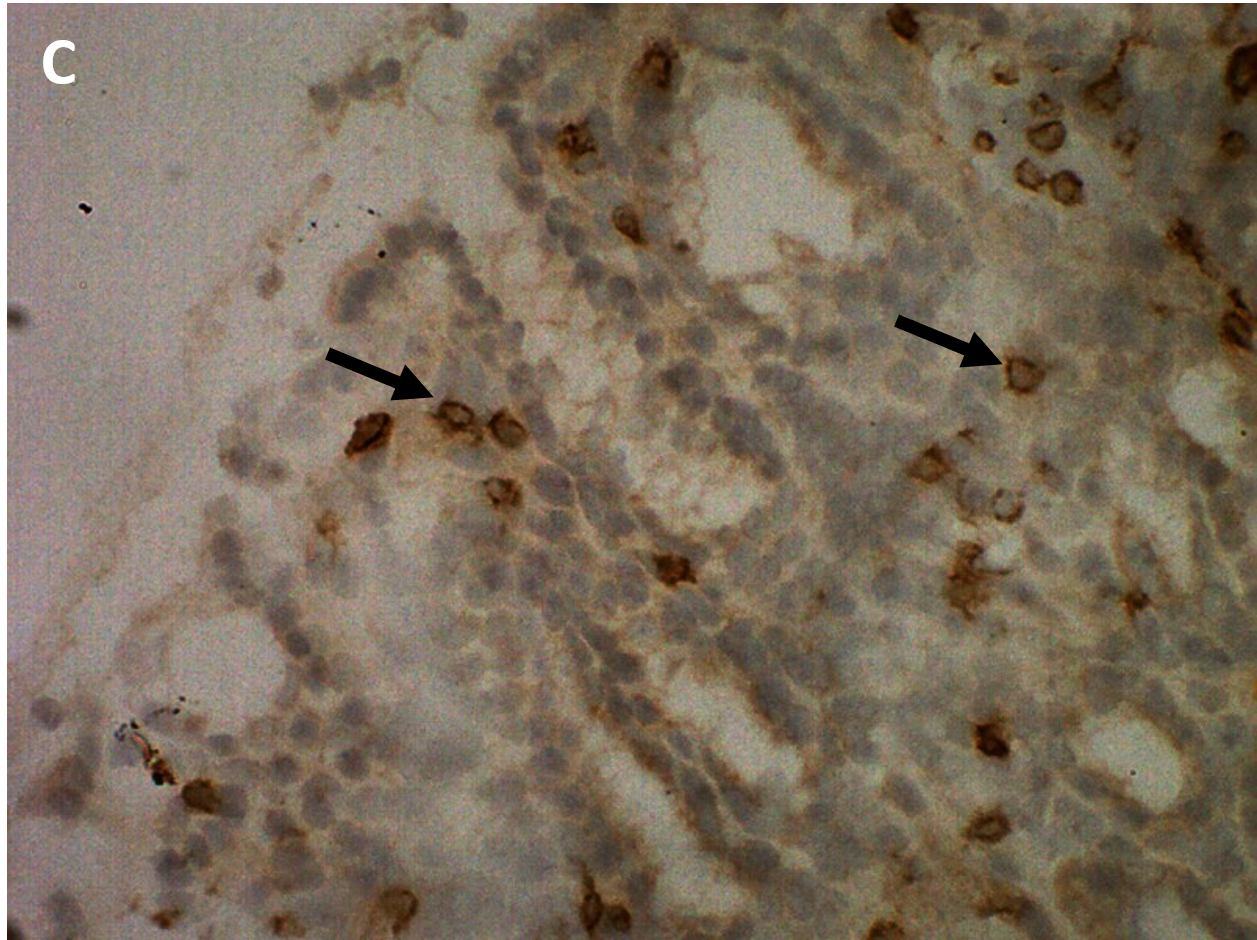


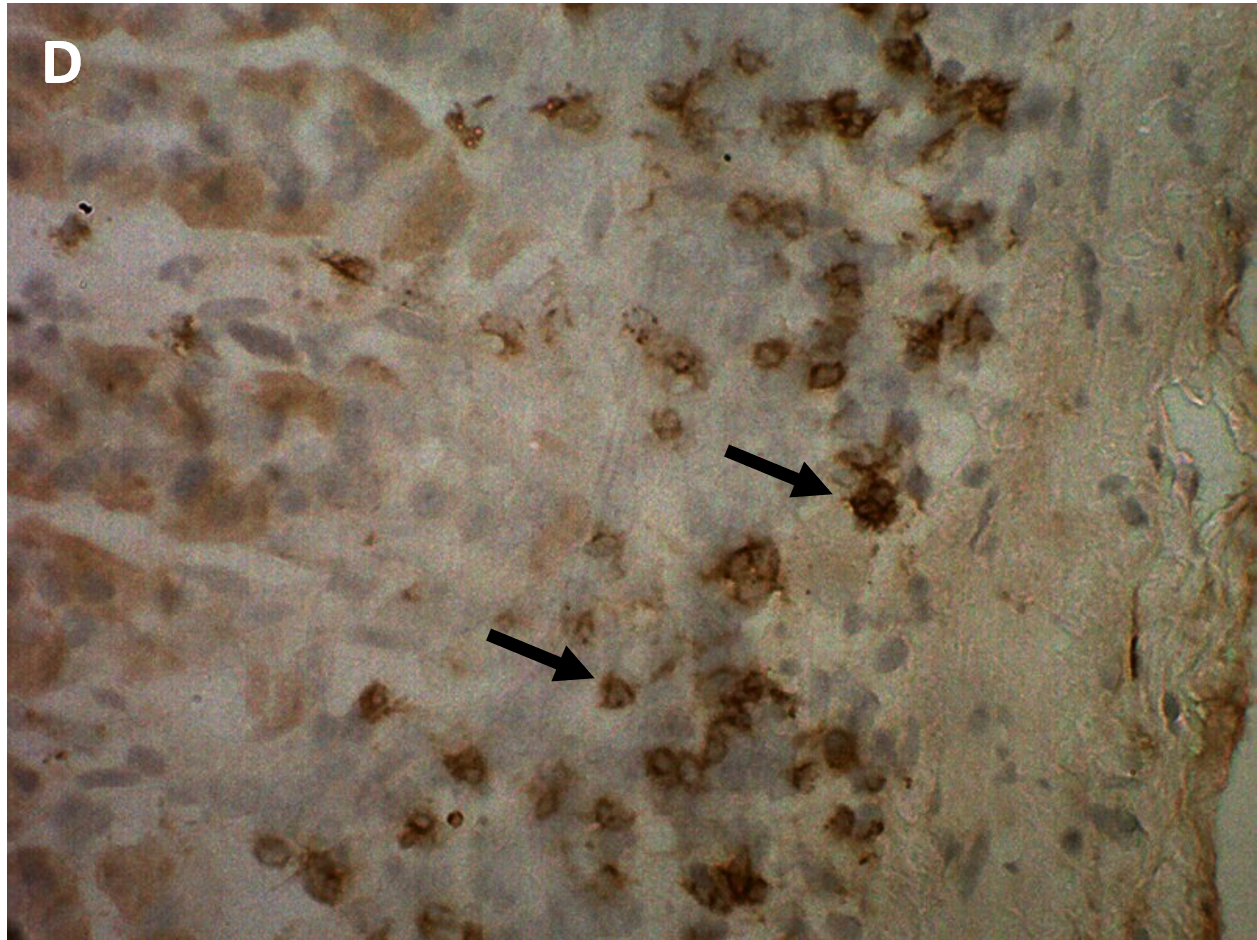

Supplement: Supplementary file 2 — Additional file 2. Examples of abomasal mucosa sections from Canaria Hair Breed and Canaria Sheep experimentally infected with Teladorsagia circumcincta showing positive cells (x400). Haematoxylin and eosin staining for globule leukocyte in the upper layer (A) and eosinophils in the basal layer (B), both indicated with arrows. Immunohistochemical staining with CD4+ antibody (SBU T4 pool 44.38 + 44.97), showing CD4+ cells (arrows) in the apical (C) and basal area (D). [file 13567_2021_960_MOESM2_ESM.docx]
