# Supplementary material for: Cellular and humoral immune responses associated with protection in sheep vaccinated against Teladorsagia circumcincta
Source: Vet Res. 2021 Jun 16;52:89. doi: 10.1186/s13567-021-00960-8 (PMC8207578; doi:10.1186/s13567-021-00960-8)
Supplement: Supplementary file 8 — Additional file 8. IgG2 against vaccine proteins and their correlation with parasitology in Canaria Hair Breed lambs. Associations are expressed as Spearman’s correlation coefficient. Same letters mean no significant differences between groups. Significant correlations are represented with “*” at p < 0.05 and “**” at p < 0.01. [file 13567_2021_960_MOESM8_ESM.docx]

|  |  |  |  | **Correlation** | | | |  |
| --- | --- | --- | --- | --- | --- | --- | --- | --- |
| **Isotype** | **Antigen** | **Group** | **Mean ± SEM** | **Cumulative FEC** | **Worm burden** | **Worm length** | **EIU** | |
| **IgG_2_** | **Tci-APY-1** | **CHB Vac** | 1.733 ± 0.050^a,c^ | -0.191 | -0.364 | -0.200 | -0.212 | |
|  |  | **CHB Con** | 0.989 ± 0.012^b^ | 0.060 | -0.063 | 0.000 | 0.140 | |
|  | **Tci-ASP-1** | **CHB Vac** | 1.701 ± 0.059^a^ | 0.164 | -0.118 | -0.067 | -0.164 | |
|  |  | **CHB Con** | 1.004 ± 0.001^b^ | 0.067 | 0.112 | 0.028 | -0.021 | |
|  | **Tci-CF-1** | **CHB Vac** | 1.844 ± 0.048^c,d^ | 0.791** | 0.409 | -0.103 | 0.600 | |
|  |  | **CHB Con** | 1.048 ± 0.014^b^ | -0.063 | -0.098 | -0.056 | -0.280 | |
|  | **Tci-ES20** | **CHB Vac** | 1.754 ± 0.048^a,c^ | 0.555 | 0.227 | -0.503 | 0.212 | |
|  |  | **CHB Con** | 1.019 ± 0.004^b^ | -0.388 | -0.081 | -0.312 | -0.632* | |
|  | **Tci-MEP-1** | **CHB Vac** | 1.891 ± 0.046^d^ | -0.100 | -0.209 | -0.467 | -0.600 | |
|  |  | **CHB Con** | 1.036 ± 0.009^b^ | -0.301 | 0.021 | -0.287 | -0.406 | |
|  | **Tci-MIF-1** | **CHB Vac** | 1.569 ± 0.078 | -0.282 | -0.191 | -0.212 | -0.030 | |
|  |  | **CHB Con** | 1.022 ± 0.005^b^ | -0.312 | -0.084 | -0.242 | -0.389 | |
|  | **Tci-SAA-1** | **CHB Vac** | 1.692 ± 0.067^a^ | 0.264 | 0.282 | 0.321 | -0.006 | |
|  |  | **CHB Con** | 1.016 ± 0.002^b^ | 0.079 | 0.186 | 0.155 | 0.014 | |
|  | **Tci-TGH-2** | **CHB Vac** | 1.709 ± 0.061^a^ | 0.327 | 0.009 | -0.042 | -0.091 | |
|  |  | **CHB Con** | 1.028 ± 0.005^b^ | 0.187 | -0.109 | 0.282 | 0.278 | |
