# Supplementary material for: Cellular and humoral immune responses associated with protection in sheep vaccinated against Teladorsagia circumcincta
Source: Vet Res. 2021 Jun 16;52:89. doi: 10.1186/s13567-021-00960-8 (PMC8207578; doi:10.1186/s13567-021-00960-8)
Supplement: Supplementary file 9 — Additional file 9. Correlations between cells and parasitological variables in Canaria Sheep lambs. Associations are expressed as Spearman’s correlation coefficient. Significant correlations are represented with “*” at p < 0.05. [file 13567_2021_960_MOESM9_ESM.docx]

| **Cell** | **Group** | **Cumulative FEC** | **Worm burden** | **Worm length** | **EIU** |
| --- | --- | --- | --- | --- | --- |
| **Eosinophils** | **CS Vac** | -0.056 | 0.273 | 0.021 | 0.021 |
|  | **CS Con** | 0.018 | -0.236 | -0.291 | -0.555 |
| **Mast cells** | **CS Vac** | -0.406 | -0.378 | -0.224 | -0.308 |
|  | **CS Con** | 0.045 | 0.609* | -0.082 | 0.045 |
| **CD4^+^** | **CS Vac** | 0.392 | -0.105 | 0.245 | 0.140 |
|  | **CS Con** | -0.273 | 0.139 | 0.067 | 0.527 |
| **CD8^+^** | **CS Vac** | -0.119 | -0.007 | -0.217 | -0.238 |
|  | **CS Con** | -0.473 | -0.200 | -0.709* | -0.491 |
| **γδ^+^** | **CS Vac** | 0.175 | 0.469 | 0.084 | 0.112 |
|  | **CS Con** | -0.300 | -0.409 | -0.300 | 0.009 |
| **CD45RA^+^** | **CS Vac** | 0.126 | 0.119 | 0.098 | 0.000 |
|  | **CS Con** | -0.218 | -0.127 | -0.109 | 0.300 |
| **MHCII^+^** | **CS Vac** | -0.014 | 0.336 | 0.126 | -0.056 |
|  | **CS Con** | 0.036 | 0.327 | 0.218 | 0.418 |
| **Galectin-14^+^** | **CS Vac** | -0.119 | -0.056 | -0.259 | -0.483 |
|  | **CS Con** | 0.018 | -0.250 | -0.152 | -0.171 |
