# Supplementary material for: Cellular and humoral immune responses associated with protection in sheep vaccinated against Teladorsagia circumcincta
Source: Vet Res. 2021 Jun 16;52:89. doi: 10.1186/s13567-021-00960-8 (PMC8207578; doi:10.1186/s13567-021-00960-8)
Supplement: Supplementary file 10 — Additional file 10. Correlations between cells and parasitological variables in Canaria Hair Breed lambs. Associations are expressed as Spearman’s correlation coefficient. [file 13567_2021_960_MOESM10_ESM.docx]

| **Cell** | **Group** | **Cumulative FEC** | **Worm burden** | **Worm length** | **EIU** |
| --- | --- | --- | --- | --- | --- |
| **Eosinophils** | **CHB Vac** | -0.527 | -0.436 | -0.067 | -0.236 |
|  | **CHB Con** | -0.347 | -0.524 | -0.056 | -0.203 |
| **Mast cells** | **CHB Vac** | -0.564 | -0.255 | -0.406 | -0.382 |
|  | **CHB Con** | -0.403 | -0.517 | 0.070 | -0.336 |
| **CD4^+^** | **CHB Vac** | 0.345 | -0.042 | 0.167 | 0.250 |
|  | **CHB Con** | -0.056 | -0.196 | -0.119 | -0.007 |
| **CD8^+^** | **CHB Vac** | -0.427 | -0.145 | 0.333 | -0.103 |
|  | **CHB Con** | 0.200 | 0.049 | -0.098 | 0.434 |
| **γδ^+^** | **CHB Vac** | -0.418 | -0.427 | 0.079 | -0.406 |
|  | **CHB Con** | -0.357 | -0.559 | -0.182 | -0.357 |
| **CD45RA^+^** | **CHB Vac** | 0.009 | -0.455 | 0.321 | 0.248 |
|  | **CHB Con** | 0.056 | 0.217 | 0.126 | 0.287 |
| **MHCII^+^** | **CHB Vac** | 0.209 | 0.173 | 0.515 | 0.333 |
|  | **CHB Con** | 0.441 | 0.476 | 0.490 | 0.524 |
| **Galectin-14^+^** | **CHB Vac** | -0.005 | -0.424 | -0.608 | -0.462 |
|  | **CHB Con** | -0.438 | -0.503 | -0.266 | -0.203 |
