# Supplementary material for: Cellular and humoral immune responses associated with protection in sheep vaccinated against Teladorsagia circumcincta
Source: Vet Res. 2021 Jun 16;52:89. doi: 10.1186/s13567-021-00960-8 (PMC8207578; doi:10.1186/s13567-021-00960-8)
Supplement: Supplementary file 11 — Additional file 11: Interleukin-17A secretion by abomasal lymph node lymphocytes following stimulation with Teladorsagia circumcincta L4 or adult somatic antigen from Canaria Hair Breed and Canaria Sheep vaccinated with a prototype recombinant sub-unit T. circumcincta vaccine and subsequently challenged with T. circumcincta. IL-17A secretion was examined in supernatants collected 4 days post-stimulation of abomasal lymph node lymphocytes with 5 µg/mL of T. circumcincta L4 or adult somatic antigen. In general, antigen-specific IL-17A release was not detected in any of the ALN cultures. N/D IL-17A were not detected, N/S no sampled availability. [file 13567_2021_960_MOESM11_ESM.docx]

| **Group** | **Animal number** | **Media only** | **ConA** | **L4** | **Adult** |
| --- | --- | --- | --- | --- | --- |
| CS Vac | 93327 | N/D | 45,2416451 | N/D | N/D |
| CS Vac | 93331 | N/S | N/S | N/S | N/S |
| CS Vac | 93333 | N/D | 76,2874025 | N/D | N/D |
| CS Vac | 93335 | N/D | N/D | N/D | N/D |
| CS Vac | 93336 | N/D | 85,1242285 | N/D | N/D |
| CS Vac | 93338 | N/D | 74,2732136 | N/D | N/D |
| CS Vac | 93340 | N/D | N/D | N/D | N/D |
| CS Vac | 93341 | N/D | 180,065352 | N/D | N/D |
| CS Vac | 93342 | N/D | 58,6632501 | N/D | N/D |
| CS Vac | 93344 | N/D | N/D | N/D | N/D |
| CS Vac | 93347 | N/D | 27,5775765 | N/D | N/D |
| CS Vac | 93348 | 38,5814849 | 28,170608 | N/D | N/D |
| CS Con | 93324 | N/D | 52,0909778 | N/D | N/D |
| CS Con | 93325 | N/S | N/S | N/S | N/S |
| CS Con | 93326 | 4,28015129 | 286,770137 | N/D | 55,6419668 |
| CS Con | 93328 | N/D | N/D | N/D | N/D |
| CS Con | 93329 | 0,25177361 | 280,72757 | N/D | N/D |
| CS Con | 93330 | N/S | N/S | N/S | N/S |
| CS Con | 93332 | N/D | N/D | N/D | N/D |
| CS Con | 93334 | N/D | 20,0072614 | N/D | N/D |
| CS Con | 93337 | 6,79788735 | 36,0036256 | N/D | N/D |
| CS Con | 93339 | N/D | N/D | N/D | N/D |
| CS Con | 93343 | N/D | 197,36893 | N/D | N/D |
| CS Con | 93346 | 251,698258 | 4,89923617 | N/D | N/D |
| CHB Vac | 93352 | N/D | 169,611108 | N/D | N/D |
| CHB Vac | 93353 | N/S | N/S | N/S | N/S |
| CHB Vac | 93356 | N/D | 860,31041 | N/D | N/D |
| CHB Vac | 93359 | N/D | N/D | N/D | N/D |
| CHB Vac | 93362 | N/S | N/S | N/S | N/S |
| CHB Vac | 93365 | N/D | 4,7836985 | 3,27305687 | N/D |
| CHB Vac | 93368 | N/D | 38,6602812 | N/D | N/D |
| CHB Vac | 93369 | N/D | 43,0786979 | N/D | N/D |
| CHB Vac | 93370 | N/D | 215,266433 | N/D | N/D |
| CHB Vac | 93372 | N/D | N/D | N/D | N/D |
| CHB Vac | 93373 | N/D | 106,164657 | N/D | N/D |
| CHB Con | 93349 | N/D | 22,8911909 | N/D | N/D |
| CHB Con | 93350 | N/D | 86,3490375 | N/D | N/D |
| CHB Con | 93351 | N/D | 60,1738917 | N/D | N/D |
| CHB Con | 93354 | N/D | 53,7472202 | N/D | N/D |
| CHB Con | 93355 | N/S | N/S | N/S | N/S |
| CHB Con | 93357 | N/D | 64,7058166 | N/D | N/D |
| CHB Con | 93358 | N/D | N/D | N/D | N/D |
| CHB Con | 93361 | N/D | 38,0318212 | N/D | N/D |
| CHB Con | 93363 | N/D | 125,131482 | N/D | N/D |
| CHB Con | 93364 | N/D | 24,5162759 | N/D | N/D |
| CHB Con | 93367 | N/D | 62,5452225 | N/D | N/D |
| CHB Con | 93371 | N/D | 118,194073 | N/D | N/D |
